# Long-term efficacy and safety of dasatinib in patients with chronic myeloid leukemia in accelerated phase who are resistant to or intolerant of imatinib

**DOI:** 10.1038/s41408-018-0122-3

**Published:** 2018-09-03

**Authors:** Oliver Ottmann, Giuseppe Saglio, Jane F. Apperley, Christopher Arthur, Eduardo Bullorsky, Aude Charbonnier, John F. Dipersio, Hagop Kantarjian, Hanna Jean Khoury, Dong-Wook Kim, Diane Healey, Lewis Strauss, Jorge E. Cortes

**Affiliations:** 1 Cardiff University, Cardiff, Wales UK; 2 University of Turin, Turin, Italy; 3 Imperial College, London, UK; 4 Royal North Shore Hospital, Sydney, NSW Australia; 5 Hospital Britanico, Buenos Aires, Argentina; 6 Institut Paoli-Calmettes, Marseille, France; 7 Washington University School of Medicine, St. Louis, MO USA; 8 The University of Texas MD Anderson Cancer Center, Houston, TX USA; 9 Winship Cancer Institute, Emory University, Atlanta, GA USA; 10 Catholic Hematology Hospital, Seoul St. Mary’s Hospital, Leukemia Research Institute, The Catholic University of Korea, Seoul, South Korea; 11 Bristol-Myers Squibb, Princeton, NJ USA

Treatment with a frontline BCR-ABL1 tyrosine kinase inhibitor (TKI; e.g., imatinib, dasatinib, and nilotinib) allows patients with chronic myeloid leukemia (CML) in chronic phase (CP) to achieve a near normal life expectancy^1^, whereas treatment for CML in accelerated phase (AP) is more problematic. While reports describe outcomes for patients with CML-AP at initial diagnosis^2,3^, outcomes have been historically worse once CP disease has progressed to AP. Approximately 50% of patients with CML-AP who receive imatinib as initial treatment develop imatinib resistance^4^ and experience disease progression^5^. Second-generation TKIs are indicated for patients with CML-CP or advanced CML resistant to/intolerant of prior therapy (including imatinib)^6^. After initial approval of dasatinib twice a day (BID) for the treatment of patients with CML resistant to/intolerant of imatinib in all stages, this phase 3 CA180-035 study (NCT00123487) was developed to investigate once (QD) or twice (BID) a day dasatinib treatment in patients with CML-AP, CML in blast phase, or Ph+ acute lymphoblastic leukemia resistant to/intolerant of imatinib.

Patients were randomized to receive dasatinib at either 70 mg BID (the standard dose at the time) or 140 mg QD. Comparison of major hematologic response (MaHR) between the dosage arms was the primary objective; MaHR included either a complete hematologic response (CHR) or no evidence of leukemia. Data from the 1-year and 2-year study reports (patients with 6.5 and 15 months’ median follow-up, respectively) noted that patients with CML-AP in the QD or BID arms obtained similar MaHR and major cytogenetic responses^4,7^. Patients who did not progress, die, or withdraw consent were followed for at least 7 years from the study start. Following the primary analysis after 2 years of the study, long-term safety outcomes in patients with clinical benefit who remained on study became the primary objective. Subsequently, efficacy, as defined by MaHR, was assessed 5 years after the study start, and safety data and date/cause of death were collected out to 7 years. The majority of the patients who remained on study at 2 years were in the CML-AP cohort (110/128; [86%]). Long-term efficacy and safety data, beyond 2 years, had not previously been reported in these patients with CML-AP, warranting additional follow-up.

As previously described, patients were diagnosed with CML-AP based on the standard definition (i.e., hematologic criteria^4^ or clonal evolution); patients with progression of a prior CML-AP diagnosis after achieving a hematologic response were also eligible^4^. Clonal evolution included additional chromosomal abnormalities besides the Ph chromosome (e.g., +8, +19, iso17q)^8^. After 2 years, patients who experienced-specific AEs (e.g., any-grade recurring fluid retention, including pleural and/or pericardial effusion, or any-grade gastrointestinal bleed despite dose reduction by one level) were allowed to switch from BID to QD dosing, but data were analyzed based on the initial randomization arm. After 2 years, 28 of 57 patients with CML-AP switched from BID to QD dosing and 6 patients switched from QD to BID dosing (Supplementary Table 1), limiting comparisons between the dosage arms made after 2 years.

Of 611 randomized patients, 317 (52%) were diagnosed with CML-AP at baseline and form the focus of this report. Forty (13%) patients continued to receive the treatment beyond 5 years. The most common reasons for discontinuation were study-drug toxicity (QD: 29%; BID: 36%) or disease progression (QD: 27%; BID: 23%). The median duration of dasatinib for patients with CML-AP was 15 months (QD) or 13 months (BID) at the 5-year cutoff (Table 1). At 7 years, the average daily dose of dasatinib was similar for patients enrolled based on hematologic criteria, clonal evolution, or prior diagnosis of CML-AP (Table 1).

**Table 1:** Dosages and duration of therapy at 7 years and survival outcomes at 5 years based on CML-AP diagnosis

	Hematologic status		Clonal evolution		Prior AP diagnosis		All patients diagnosed with CML-AP	
	QD (*n* = 67)	BID (*n* = 64)	QD (*n* = 55)	BID (*n* = 61)	QD (*n* = 34)	BID (n = 31)	QD (*n* =1 57)	BID (*n* = 160)
Average daily dose, mg (range)	119 (26–216)	108 (13–178)	94 (20–162)	97 (22–171)	106 (34–174)	87 (21–206)	NR	NR
Median duration of therapy, months (range)	8.3 (0.0–90.1)	9.2 (0.5–86.1)	21.9 (0.5–92.9)	20.7 (0.4–84.6)	16.9 (0.12–93.2)	20.3 (1.6–83.8)	15.4^a^ (0.00–67.0)	12.5^a^ (0.4–66.3)
Median duration of therapy excluding interruption, months (range)	7.4 (<0.1–89.1)	8.3 (0.5–85.4)	20.9 (0.5–92.9)	16.7 (0.3–83.7)	15.4 (0.2–91.4)	17.3 (1.6–81.7)	NR	NR
PFS, % (95% CI)	20.7 (11.5–31.9)	21.3 (10.2–35.2)	42.1 (27.4–56.1)	41.5 (25.0–57.2)	31.1 (15.8–47.8)	24.0 (7.1–46.1)	30.5 (22.6–38.5)	30.3 (20.9–39.6)
OS, % (95% CI)	32.8 (21.1–44.9)	45.6 (32.3–58.0)	52.6 (37.9–65.3)	67.9 (53.5–78.7)	54.3 (35.9–69.4)	60.2 (40.4–75.2)	45.0 (36.8–53.2)	57.6 (49.5–65.7)

*AP* accelerated phase, *BID* twice a day, *CI* confidence interval, *CML* chronic myeloid leukemia, *NR* not reported, *OS* overall survival, *PFS* progression-free survival, *QD* once a day

^a^Duration of therapy for entire CML-AP population calculated at 5 years

The rate of MaHR by 5 years (QD: 67%; BID: 69%) was consistent with the 15-month report^4^, suggesting that most patients may have reached maximum response by 15 months. The median duration of response in patients with MaHR was 54 months at 5 years for the QD arm and 55 months for the BID arm (Fig. 1). Progression due to loss of hematologic response slightly increased in both the QD and BID dosage arms from 12% and 14%, respectively, at 2 years to 15% in both arms at 5 years. The best response of CHR at any time within 5 years was reached in 51% and 54% of patients in the QD and BID arms, respectively.

**Fig. 1:**
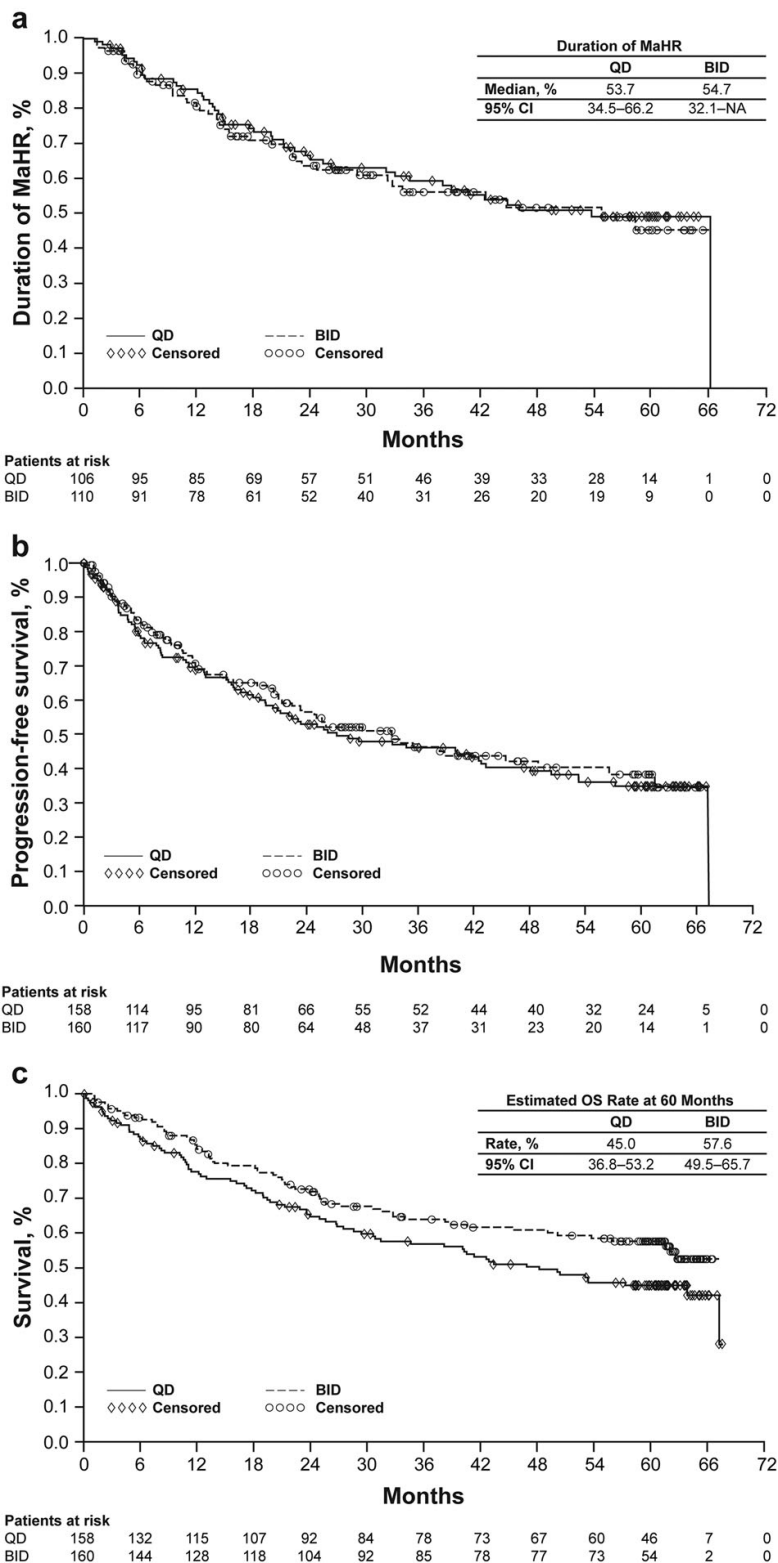
Efficacy outcomes for patients with CML-AP by dosing schedule. **a** Duration of MaHR for patients who achieved MaHR, **b** PFS, and **c** OS are shown for the QD (solid lines) and BID (dashed lines) dosing groups. Efficacy analyses were conducted in the intention-to-treat population of patients. *BID* twice a day, *CI* confidence interval, *MaHR* major hematologic response, *NA* not available, *OS* overall survival, *PFS* progression-free survival, *QD* once a day

Dose schedule did not appear to affect 5-year rates of progression-free survival (PFS) in patients with CML-AP (Table 1 and Fig. 1). Patients with clonal evolution at enrollment had a numerically higher 5-year PFS rate (42% in both arms) compared to patients diagnosed based on hematologic status (21% in both arms) or having a prior diagnosis of CML-AP (QD: 31%; BID: 24%) (Table 1). Five-year overall survival (OS) rates were numerically higher in patients who were assigned BID versus QD dosing (Fig. 1) whether diagnosis was based on hematologic status (46% vs. 33%), clonal evolution (68% vs. 53%), or prior diagnosis of AP (60% vs. 54%) (Table 1). Although 5-year OS rates were higher for the BID versus QD arm, the hazard ratio (HR; 1.37 [confidence interval: 0.99–1.90]) suggests comparable effects.

After 7 years of follow-up, 83 of 157 (53%) and 67 of 160 (42%) patients had died in the QD and BID arms, respectively, 25 (15%) and 17 (11%) within 30 days of their last dasatinib dose. Only one patient died due to study-drug toxicity; the patient was enrolled into the QD arm and died at <2 years on study.

Data relating to the presence or absence of mutations at baseline and at the end of treatment/disease progression were available for a total of 61 (QD) and 58 (BID) patients at 5 years. The treatment arms had a similar proportion of patients with no identified mutations at baseline (QD: 59% BID: 52%) and at the end of treatment (QD: 49%; BID: 50%). Over the course of 5 years, 16 patients (QD: 10; BID: 6) with no mutation at baseline had a new mutation identified. The most common acquired mutations were T315I (QD: 9; BID: 10) and F317L (QD: 5; BID: 4). Conversely, of patients with an identified baseline mutation (QD: 25; BID: 28), the mutation was no longer detected at 5 years in nine patients (QD: 4; BID: 5). The most frequently lost mutations were E255K in the BID arm (*n* = 5) and M351T in the QD arm (*n* = 4).

With respect to treatment-related AEs after 5 years of follow-up (Supplementary Fig. 1), any-grade treatment-related fluid retention events occurred more frequently in the BID (53%) versus QD (40%) arm. Treatment-related pleural effusion events of any grade slightly increased in incidence between 2 years (QD: 31 [20%]; BID: 62 [39%]) and 5 years (QD: 43 [27%]; BID: 71 [44%]); grade 3–5 pleural effusion occurred in 14 (9%) and 15 (9%) patients in the QD and BID arms, respectively, at 5 years. While the incidence of pleural effusion was unchanged between 5 and 7 years in this study population, others have reported a continuing risk over time after years of dasatinib treatment^9^.

At 5 years, there were four cases (two grade 3–5) of drug-related congestive heart failure/cardiac dysfunction in patients in the BID arm. Drug-related pulmonary hypertension was diagnosed by echocardiogram in three patients and indicated by X-ray in one case; it was observed in one (grade 3–5) and three (grade 1–2) patients in the QD and BID arms, respectively, within 5 years. The case in the QD arm was diagnosed on study day 944 and the cases in the BID arm were diagnosed on study days 36, 463, and 838. There were no cases of pulmonary arterial hypertension (PAH); however, right heart catheterization is required to confirm PAH, and only one procedure was performed.

The frequency of hematologic and biochemical AEs was similar between the two dosage groups and was consistent with the earlier study report^4^. No new safety signals were identified in patients who remained on treatment after the 5-year cutoff.

Data from the CA180-035 trial represent the largest study to date, with the longest follow-up, of dasatinib-treated patients with CML-AP. After 5 years of treatment, rates of MaHR, PFS, and OS in the QD and BID dosing arms suggest comparable efficacy with either regimen. Maintenance of MaHR suggests that some patients with CML-AP, who are responding well to second-line dasatinib therapy and are at high risk for transplant-related mortality may not need to seek a stem cell transplant. More AEs, specifically fluid-retention events (e.g., pleural effusion), occurred in patients with CML-AP in the BID versus QD arm. The reason for higher OS in the BID arm is unclear. However, because there is no significant difference in survival between these dosages, BID dosing has a more complicated safety profile, and patients with CML-AP who respond to and tolerate dasatinib treatment can maintain long-term responses, the data reinforce the current QD dose indication for treatment of patients with advanced CML. Overall, the 5-year efficacy and 7-year safety data in dasatinib-treated patients after imatinib intolerance/resistance presented here support use of dasatinib for long-term treatment of patients diagnosed with CML-AP.

## Electronic supplementary material

Supplementary Table 1

Supplementary Fig. 1
