# Supplementary material for: Long-term efficacy and safety of dasatinib in patients with chronic myeloid leukemia in accelerated phase who are resistant to or intolerant of imatinib
Source: Blood Cancer J. 2018 Sep 3;8(9):88. doi: 10.1038/s41408-018-0122-3 (PMC6127283; doi:10.1038/s41408-018-0122-3)
Supplement: Supplementary file 1 — Supplementary Table 1 [file 41408_2018_122_MOESM1_ESM.docx]

**Supplementary Table 1. Patients switching dosing arms between 2 and 7 years and last dose received**

|  | **Schedule at randomization** | |
| --- | --- | --- |
|  | **Patients on study following the switch amendment (n = 114)** | |
| **Patients, n (%)** | **QD (n = 57)** | **BID (n = 57)** |
| **Schedule at last dose** |  |  |
| QD | 51 (45) | 28 (25) |
| BID | 6 (5) | 29 (25) |
| **Last total daily dose, mg** |  |  |
| **QD** |  |  |
| <80 | 8 (7) | 16 (14) |
| 80 | 7 (6) | 3 (3) |
| 90 | 0 | 4 (4) |
| 100 | 12 (11) | 3 (3) |
| 140 | 15 (13) | 2 (2) |
| >140 | 9 (8) | 0 |
| **BID** |  |  |
| <80 | 1 (1) | 5 (4) |
| 80 | 2 (2) | 7 (6) |
| 100 | 0 | 8 (7) |
| 140 | 1 (1) | 4 (4) |
| >140 | 2 (2) | 5 (4) |

**Supplementary Fig. 1 Incidence of treatment-related AEs of special interest occurring in ≥10% of patients with CML-AP at 5 years.** Any-grade (solid) and grade 3–5 (patterned) AEs are displayed for both the QD (black) and BID (gray) dosage groups. *AE* adverse event, *BID* twice a day, *GI* gastrointestinal, *QD* once a day. Other hemorrhage includes bleeding other than GI bleeding or central nervous system bleeding

.
