# Supplementary figures and images for: Long-term efficacy and safety of dasatinib in patients with chronic myeloid leukemia in accelerated phase who are resistant to or intolerant of imatinib
Source: Blood Cancer J. 2018 Sep 3;8(9):88. doi: 10.1038/s41408-018-0122-3 (PMC6127283; doi:10.1038/s41408-018-0122-3)

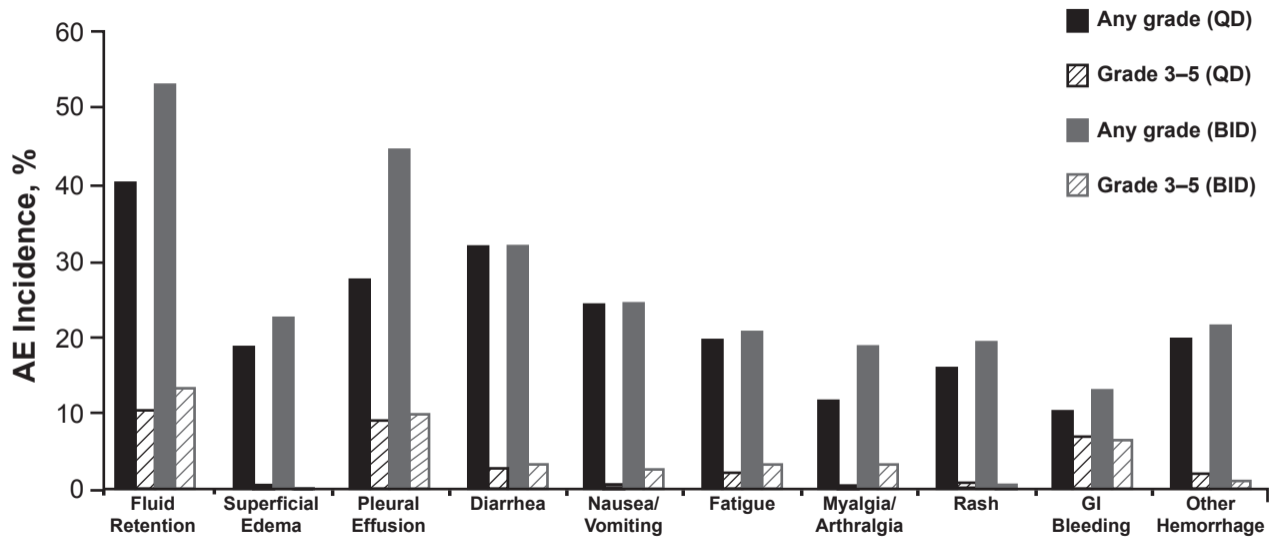

Supplement: Supplementary file 2 — Supplementary Fig. 1 [file 41408_2018_122_MOESM2_ESM.pdf]
